# Utility, barriers and facilitators to the use of connected health to support families impacted by paediatric cancer: a qualitative analysis

**DOI:** 10.1007/s00520-022-07077-4

**Published:** 2022-05-06

**Authors:** Emma Delemere, Isaiah Gitonga, Rebecca Maguire

**Affiliations:** Department of Psychology, Maynooth University, Co. Kildare, Ireland

**Keywords:** Connected health, Paediatric cancer, Attitude, eHealth, Families

## Abstract

**Aim:**

As healthcare systems are increasingly burdened, the efficiencies and cost savings offered by connected health (CH, i.e. two-way communicative healthcare technologies such as eHealth or mHealth) present an attractive solution for supporting families impacted by cancer. More research is required, however, to examine attitudes towards CH to better facilitate its use in practice. This study seeks to examine the utility, barriers and facilitators of CH use for families affected by paediatric cancer living in Ireland.

**Methods:**

Healthcare professionals (n = 5) and parents of children with cancer (n = 7) completed semi-structured interviews on their experiences of and attitudes to CH via Microsoft Teams. A reflexive thematic approach to analysis was employed.

**Results:**

CH was perceived to provide support for a number of current needs with themes of ‘shifting responsibilities’, ‘individualisation of care’ and ‘knowledge as power’. Through facilitating communication, information sharing and monitoring of child health, CH was perceived to support decreased parental burden and increased parental control, with positive child outcomes thought likely. Perceived barriers and facilitators to the use of CH included the ‘importance of trust’, ‘pace of change’ and ‘access’.

**Conclusion:**

While results suggest an acceptance of CH across key stakeholders, barriers and facilitators should be considered to support effective implementation. While further analysis of the efficacy of CH to support families impacted by paediatric cancer is needed, these findings highlight key areas where CH may be effectively employed.

**Supplementary Information:**

The online version contains supplementary material available at 10.1007/s00520-022-07077-4.

As diagnosis and survival rates for paediatric cancer increase [1], so too does demand on healthcare services designed to support those coping with cancer. As a result, healthcare providers (HCPs) are increasingly turning to technology, motivated by a need to decrease costs, while increasing access to services [2, 3]. One area of recent technological advancement is connected health (CH), a sociotechnical approach to healthcare linking people, process and technology [4]. More specifically, CH is often used as an umbrella term to describe sensor technology, mHealth and eHealth, amongst others [5, 6]. Great potential exists for CH in supporting those with serious illness [7], through reducing bottlenecks [8], facilitating communication between primary and secondary care [9], and aiding transfer of clinical information [10] through allowing for efficient collection, analysis and transfer of data through technology. While limited analysis of CH interventions within paediatric cancer has been conducted [11], such technologies offer particular benefits for this population through aiding understanding [12] and improving parent–HCP communication [13] by easing access to health information, reducing social isolation through facilitating communication with peers [12], and enhancing healthcare management through ease of access to health records [14]. However, variability exists in parent attitudes towards technology to support caregiving [15], with little work examining preferences towards service delivery approaches [16]. Additional analysis of perceptions towards the use of CH from the perspective of key stakeholders is needed to determine their utility in supporting families with childhood cancer.

As CH technologies become integrated within healthcare systems, evaluation of their efficacy is paramount. Several limitations have been noted, including privacy concerns, incompatibility with pre-existing systems [17], ethical and legal concerns [18] and generalisation of effects to non-pilot settings [19]. Further, concerns regarding reductions in the availability of face-to-face supports in response to increased digitalisation have been noted [20]. A recent systematic review of adverse effects of eHealth interventions on patient–provider relationships raised concerns regarding the impacts on patient centred care, though few studies were found [21]. Additionally, concerns regarding impacts of eHealth on workload reducing face-to-face supports were also raised.

A further factor inhibiting CH adoption is patient and provider acceptability. While mixed acceptability has been found for adult cancer patients [22], limited analysis has been conducted in paediatric cancer. One systematic review of CH for families living with or beyond childhood cancer found good acceptability and usability [19]. While this suggests acceptance of CH overall, attrition from interventions remains high [23, 24]. This also appears to be the case in paediatric cancer, with difficulties recruiting and retaining users [25]. HCPs play a vital role in CH uptake [26] supporting patient acceptance [27]. While positive HCP attitudes towards CH been found [28], so too has resistance to use [29]. Concerns regarding limitations on communication, data security, privacy and impacts on the therapeutic relationship have been raised [30]. While HCPs report positive impacts of CH on patient knowledge, quality of life and living standards, few wished to use these tools themselves [8]. While research somewhat suggests the acceptability of CH, additional analysis is needed.

One theoretical approach that may provide insight into CH uptake is the Technology Acceptance Model (TAM; [31]). The TAM posits that behavioural intention, or willingness to use a technology, is impacted by the degree to which the technology is perceived as useful and easy to use [32]. This TAM has received significant empirical analysis, with a recent meta-analysis highlighting its efficacy as a model of technology acceptance [33]. The impact of perceived usefulness and ease of use has also been demonstrated for health technology [34, 35]. Analysis of TAM within healthcare found it to effectively explain user acceptance; however, additional analysis of the unique context of healthcare is needed [36]. More recent analysis has expanded this model, with social influence [37], economic burden and data privacy also found to impact acceptance [38]. As such, for CH to be acceptable to stakeholders in paediatric cancer, exploration of how these technologies may be applied in a manner which is useful is needed, while also considering economic and privacy impacts.

This study seeks to examine the utility, barriers and facilitators of CH within an Irish context for families affected by paediatric cancer and their HCPs. Over 200 children are diagnosed with cancer in Ireland each year, with prevalence and survivorship rates rising [39]. Paediatric cancer care is delivered through a centre of excellence model, requiring families to travel significant distances for care. Increasing remote service delivery may be particularly welcomed for this group in response to this significant travel burden. There is currently an absence of digitalisation within the Irish healthcare system, with poor uptake of digital technologies. Specifically, paediatric cancer care in Ireland tends to rely on physical patient records, with little or no use of electronic health records (EHRs). A commitment to digitalisation has, however, been espoused [40]. Qualitative analysis is employed as it allowed for an in-depth exploration of stakeholder perspectives towards CH [41]

## Methodology

### Recruitment of study participants

For parents, eligibility criteria consisted of having a child (aged 0–18) with cancer at least 6 months post-diagnosis but less than 5 years from active treatment. HCPs were qualified with at least 1 years’ experience working with children with cancer. As this study was completed as part of a series of studies, participants were recruited across studies in tandem. A snowballing strategy was employed using social media platforms and circulation of recruitment invitations to professional groups and governing organisations in paediatric illness. As a reflexive thematic approach to analysis was applied and in line with past research [42, 43], data saturation was not used to determine sample size, with a focus instead on rich data acquisition. The present sample is in line with recommended minimum sample sizes to allow for meaningful analysis [44]. Of individuals approached, only one HCP declined to participate.

Full ethical approval for this study was obtained through the Maynooth University ethics board (reference number: SRESC-2020–2414528). Full informed consent was obtained through a consent form emailed to participants 3–5 days prior to the interview, which was electronically signed and returned. Verbal consent to participate at the start of each interview was also obtained. All aspects of this study were conducted in accordance with the Declaration of Helsinki [45].

### Epistemological approach

The current study employed a paradigmatic framework of interpretivism and constructivism within a phenomenological qualitative approach [44]. The focus of this research was to understand participants’ view of connected health as it pertains to their role. Specifically, this study sought to reflect parent and HCP accounts of their needs and hesitations pertaining to CH, while also accounting for the reflexive influence of the researcher on analysis. Reflexive thematic analysis [46] was selected as the most appropriate approach to analysis here as it permitted an open consideration of participant perspectives, while appreciating their subjectivity and the reflexive influence of the researchers’ own interpretations. The flexibility of the thematic analysis approach facilitated the bottom-up inductive analysis of data, though some deductive analysis was also used when considering the themes in relation to the research question and broader theoretical background. The interview process was determined by ED and RM (who has experience in qualitative research) through discussion in which research questions were drafted and biases considered using a reflective process. The researcher comes from a world view perspective of a ‘behavioural psychologist and doctoral researcher, with no experience as a HCP, as a parent or with serious childhood illness’, with an emic ontological position.

### Interview guide and data collection

A semi-structured interview format was used to facilitate open discussion and allow exploration of topics raised by participants. As such, while a set interview guide was developed, the specific wording and order was not rigidly adhered to. Interview questions can be seen in Table 1. The interview with the first participant from each group (HCPs and parents) acted as a pilot, with these participants asked to share feedback and suggestions. The feedback was used to refine the questions and probes in the subsequent interviews.

**Table 1:** Interview Guide

Connected health is defined as the use of smart technologies, like sensors, telehealth or electronic health records, within healthcare. It differs from other technologies in that a two-way flow of information is used. Information is gathered, analysed and then fed back to the individual With that in mind what potential use would CH offer parents, children and families impacted by paediatric cancer? Specifically, what unmet needs could it aid
What currently unmet needs of parents, children and families affected by paediatric cancer could CH support?
What barriers or limitations would there be to the use of CH?

Interviews were conducted via Microsoft Teams between December 2020 and April 2021. Both audio and video were recorded for most interviewees (2 HCPs used audio only). Interviews were conducted by ED (researcher), who has completed training in conducting interviews and has 5 years’ experience conducting interviews with parents of children with disabilities. Average interview duration for HCPs was 32.43 min (range 31.05–35.46 min) and 38.16 for parents (range 23.36 to 56.48 min).

### Data analysis

A reflexive thematic content analysis approach was chosen due to its flexibility and accessibility [44, 46]. While data analysis employed primarily an inductive approach, some deductive analysis was used to ensure themes were applicable to the research questions. Prior to analysis, all interviews were transcribed into Microsoft Word by ED. In an effort to reduce burden on participants in the context of COVID-19 and in line with past recommendations [47], transcripts were not shared with participants following interviews. Following this, data were analysed using a recursive approach to Braun and Clarke’s [46] six steps. Firstly, transcripts were re-read to develop good familiarity with the contents. Coding was then completed by ED (a PhD student with no past experiences with serious childhood illness) using QDA Miner Lite, with codes given to important features of data. Accuracy was confirmed by re-reading codes in the absence of data to ensure they held on their own. A sample of two transcripts across participant groups was then coded by IG (a doctoral researcher with experience in qualitative analysis). Following this, the researchers openly discussed codes and themes arising. A consensus was arrived at on the final themes used in the manuscript. Codes were then grouped and allocated according to content into themes, which were reviewed by re-reading all transcripts to ensure no data were omitted. As per Tong et al.’s. [48] consolidated criteria for reporting qualitative research, themes were not anticipated in advance and derived directly from data collected. Finally, themes were defined and named, and data were written up. While uncommon in thematic analysis [49], theme frequencies across participant groups were reported in an effort to reflect the unique experiences and contexts of participant groups and to allow for differences between them to be considered. However, these frequencies are intended to highlight shared experiences across groups only, and caution should be taken in their analysis with no additional strength in themes reflected by frequencies.

## Results

Participants consisted of parents of children with paediatric cancer (n = 7) and HCPs (n = 5; one nurse, two doctors, one social worker and a physiotherapist). HCPs were primarily female (n = 4) and had an average of 17.6 years’ experience. Parents were 38.8 years old on average, were primarily female (n = 6), married (n = 3) or cohabitating (n = 3) and lived in small towns (n = 6). Mean age of children was 8 (range = 4–12), and most had siblings (n = 5; mean siblings = 2.6, range 1–4). Children were primarily diagnosed with acute lymphocytic lymphoma (n = 2) and rhabdomyosarcoma (n = 2), and most had finished active treatment (n = 5).

### Themes

Six themes were noted, of which three pertained to potential areas of need which CH may support, and three which described facilitators or barriers that may impact CH use. ‘Shifting Responsibilities’, ‘Individualisation of Care’ and ‘Knowledge as Power’ were perceived as needs, which could be addressed by CH, while ‘Importance of Trust’, ‘Pace of Change’ and ‘Access’ were noted as barriers and facilitators of CH. Table 2 includes additional details and illustrative quotes.

**Table 2:** Challenges and Needs of families

Area	Themes and sub-themes	Number of participants who mentioned the theme		Illustrative Quotes
		Parent	HCP	
Needs	1) Shifting Responsibilities	7	5	1. “I suppose from the starting out again when you’re on your journey, and fair enough when you being and they’re assessing everything, but like yknow meeting the intern, consultant at the start and starting the story again and yknow it’s the middle of the night you haven’t slept in a day or two and you’ve been at work and to start from the start is very upsetting” P6 2. “Every time you go into hospital it’s almost like you’re doing mastermind on the treatment like literally printed sheet you could just hand over so when you go, cos you could be in and out of hospital constantly” P3 3. “A lot of the time HOSPITAL are saying oh yeah we’ll have to ring Dublin for that or we’ll have to, yknow. There’s definitely a space for sharing that information in a far more efficient way” p7 4. “You’re ringing and ringing and ringing for results …. Like when a report is ready it should be ready when its ready for the oncologist it should be ready for the parent I don’t see why not.” P4 5. “I mean its really important that technology would help us be very accurate yknow the parents would get rather than have to write things down on paper, that they’d get a print out of the child’s blood results….Like that kind of thing should be done to make information available to parents” HCP5
	2) Individualisation of Care	6	3	1. “There’s work with a Swiss group that have used sort of high-tech Fitbit like things to look at heart rate blood pressure temperature, changes from baseline rather than absolute temperatures and so on to see if that would give us an early warning of of adverse consequences coming on and potentially really serious adverse consequences coming on… and the idea being that that might lead on to us being able to intervene em before the infection took hold for example and give some sort of treatment that would be less and lower level and keeping the patient well-er without being in hospital for so long.” HCP1 2. “If we knew what their ideal target amount of chemo was, and we know of the variation between people maybe we could get the blood monitored in a really regular and straight forward way and alter the dosing for that person and maybe that would then get us the most anti-canceriness without getting the most, without getting the side effects that go with it” HCP1 3. “I know our physiotherapists who deal with our patients are very keen to try and promote physical activity. And maybe that is someway tangently to start is to monitor physical activity in the community and when they come and see us in the clinic just look at their electronic footprint of activity and see can we improve it” HCP2 4. “I remember thinking oh god wouldn’t it be great to have an app to be record these things so we could build a picture cos there was a cycle of sick, yknow like she would be ok for a while and then there was a dip and that would be when she would be in her neutropenic phase and you had to be so careful” P4
	3) Knowledge as Power	7	3	1. “Where to get information from I think that’s kind of half the battle” P2 2. “We’d have conversations with the consultant but like my mind was just I still have memory loss from it I swear somethings gone in my brain. … And even, me and FATHER used to remember different bits of conversations, so maybe like a summary you could see electronically of the conversation you had” P3 3. “I would often ask the doctors can I see, can I see his ultrasounds can I see his MRIs, and I’d ask to compare to the last one. I think I think it’s needed, cos I think when you can’t see something that’s going on you can’t fully understand it” P5 4. “Tracking eh symptoms definitely that would be useful especially from the beginning when you are not used to everything. When if he has a temperature between this range then you should bring him in and that was a big struggle because now I know from the top of my head when I should bring him in or not, but back then it was all new” P1
Facilitators and Barriers	1) Importance of Trust 1a) Privacy	3	4	1. “I can’t see anyone being held to ransom over the fact their blood count was a haemoglobin of 73 or whatever. But I I understand the the fear of other people being able to see other things about me that I don’t want them to know” HCP1 2. “If there is a GDPR breach there is a GDPR breach, so who wants to know NAME’s neutrophils like.” P6
	1b) Monitoring and Accuracy	4	4	1. “I wouldn’t have concerns just yknow just I suppose that its validated so like yknow… that records are kept accurately. So, somebody isn’t acting, that there is a bit of triangulation.” P6 2. “We’d need to make sure what information we’re expecting of them, how we’re measuring it and how accurate it is and then what we’re going to do with it.” HCP4 3. “There’s no point like getting emails that come in and we don’t look at them for 24 h and somebody’s email is saying they’re unwell. Those things would concern me.” HCP4
	1c) Recommendations for use	3	0	1. “I remember they tell you not to go on, only look at these sites, you need information if you can’t quite find the information on that one, then you end up googling it and you end up on the bad sites that you’re not supposed to read. And it’s this whole misinformation really,”P3 2. “I would always look to the to the hospital, it’s like right if they recommend then I would be happy enough to do it but if something popped up on my whatever social media to say awh you can use this app or use this for id still be fairly wary of it” P2
	2) Pace of Change	6	5	1. “We are not em user friendly with modern IT patient interactive bits, em I I think we could be a little bit better in that but we are putting our thrust in the electronic patient record going forward” HCP2 2. “No. never heard of it. I was only saying to NAME she is going to talk to me about Connected Health and I’ve no idea what that is, should I have an idea of what that is?” P5 3. “We’re collaborating with NAME with the redcap database, and it’s taken 2, 3 years to get to this point where we are now, ready to go.” HCP2 4. “I do wonder a bit whether it’s partly it worked much better than people expected. It’s probably better to do lots of things in person but everything’s got a price and the price of getting your kid to a group for them to be part of it if your geographically disparate, versus being able to do it over a zoomy thing, so yknow I can see how things will maybe shake out a little differently than before” HCP1
	3) Access 3a) Facilitate Access	5	3	1. “Some of our patients can travel 4–5 h to get to us. And we may not need to see each patient in the clinic every time. We may be able to do it virtually and therefore maybe every second visit then can come to Dublin for their interaction.” HCP2 2. “There is something floating around that’s been talked about a lot. And that’s having the ability to almost do your own blood tests at home so you wouldn’t even need to have the blood sent somewhere to be counted.” HCP1 3. “You are conscious of, you know, infection if your child has an infection, everyone is immunocompromised so if there was like a parents support online at a suitable time that we don’t have the added stress of struggling somewhere to meet physically” P1 4. “They had mentioned something about being able to do the neutrophils at home, and it’s like electronic like what you’d have I suppose for like for blood sugars, you know you take with the prick. And I was like that to me would’ve been amazing when we were doing it, so you’d just be aware of how her immune system, how her neutrophils, how things are yknow whether shed be able to go maybe try going somewhere” P4 5. “Having the ability for kids even in isolation or even young people in isolation to get together and go around things and that in itself with the online gaming communities is certainly a way that many teens, maybe more boys than girls, but many teens stay connected to their peer group” HCP1
	3b) Reduce Access	4	5	1. “From a financial point of view some bits of equipment can be quite expensive and childhood cancer, having a diagnosis of childhood cancer can have quite financial burden on families… And so some families may not be in a position to purchase equipment” HCP3 2. “Not all parents are able to read or write. Em and so I know some platforms obviously can have built in things I suppose to dictate and read out what’s on screens but that could be another potential barrier” HCP3 3. “Well if you think of family who weren’t in a good broadband area or some people don’t have technology em they might not have a phone even you know” P4

### Needs CH may support

#### Shifting responsibilities

The potential for CH to shift communicative responsibilities was noted, particularly by parents. When meeting with HCPs, parents were often required to recall information on child health. This requirement to re-tell your child’s story was seen as a source of stress, with concerns over the impact an error or omission may have on their child’s care. Parents felt CH may alleviate this by providing a single source of information, which could be updated and accessed by multiple professionals.

Potential for CH to aid communication between HCPs was also posited. Often children have large medical teams requiring frequent transfer of information between disciplines or healthcare settings. While information was shared using paper files, parents were frequently relied upon to share information across HCPs. Rather than parents having to directly seek or share information, seen as an “activation bump” (HCP1), CH could allow for more free and timely transfer of information.

While CH may facilitate communication, the importance of supplementing, rather than replacing, face-to-face communication was noted. This was emphasised for disciplines relying on interpersonal connection, particularly psychology and social work services.

#### Individualisation of care

The potential benefits of individualising healthcare were expressed, particularly by HCPs. Through more systematic and comprehensive tracking of child health, CH could allow more timely responses to infections or adverse consequences to be made. HCPs queried whether ongoing monitoring and analysis of blood or other measures using CH could facilitate more individualised protocols, while reducing side effects. For outpatient care, support to monitor treatment adherence and progress was highlighted, facilitating individualised future recommendations.

#### Knowledge as power

Parents expressed the importance of a single source of trusted information to facilitate ongoing knowledge exchange. Parents frequently sought additional information on their child’s health to increase their understanding and to aid in care provision. Difficulties identifying trusted sources of information were noted. While parents were provided with information by HCPs verbally, they felt this too was insufficient. Difficulties remembering information shared during conversations with HCPs were reported, often due to the high stress and volumes of information shared. As a result, questions would often arise following the appointment. Additionally, terms used by HCPs were sometimes difficult to follow. Support to visualise their child’s diagnosis and progress in treatment was sought, with access to X-rays or scans felt to be a more accessible means for parents. In addition to understanding, information to support decision-making by parents was also needed, particularly in determining when actions should be taken regarding their child’s health.

### Factors impacting CH uptake: Facilitators and barriers

#### Importance of trust

The importance of trust when considering the use of CH was noted. This included trust in data privacy, in the quality of the system, and that data were being appropriately monitored.

##### Privacy

For HCPs, ensuring that any system was secure was a key consideration for use. HCPs concerns pertained more, however, to alleviating parental concerns, rather than fear of harm. For parents, security of data was a key consideration. Again, a perceived low risk of harm should privacy be breached was noted.

##### Monitoring/accuracy

For systems such as EHRs, where multiple HCPs may be accessing information, ensuring data remained up to date was a key priority. Additionally, HCPs noted a need to ensure accuracy, particularly where parents were inputting or monitoring data. HCPs also raised concerns regarding the monitoring of data inputted into CH. As data inputted may require action on the part of healthcare teams, effective monitoring and response protocols are needed.

##### Recommendations for use

The need for trusted professionals or HCPs to act as gatekeepers to CH was noted to aid trust in the technology. Parents reported cynicism towards online sources of health information with inappropriate or inaccurate content common. To mitigate this risk and to facilitate use and trust of a CH system, a referral from a trusted source, such as a HCP, is needed.

#### Pace of change

At present, there appears to be an absence of technology within service provision, with a conservative approach taken to technology introduction. HCPs noted a reliance on paper to manage information, though this was an area of upcoming change. While HCPs were highly aware of the many CH tools, which could support service delivery, a disbelief in their introduction in the short term was noted, alongside an acceptance of the slow pace of change. For those HCPs who had participated in digitalisation efforts, the pace of introduction was felt to be slow and hard fought. COVID-19 was thought to have had a positive impact on the use of technology in health, with many previously in-person services forced online, often successfully.

#### Access

Access to services was felt to be both positively and negatively impacted by CH. CH was seen as an avenue to reduce the response effort to access services, increase access to one’s own community and provide social support for children. However, cost, access to WI-FI and literacy were seen as means through which CH may limit service access.

##### Increase access

Both groups noted the potential for CH to increase access to services through reducing the response effort required. As healthcare services for children with cancer in Ireland are delivered through a central children’s hospital in a large urban area, families travel long distances to access treatment. CH may reduce some of this travel through allowing for monitoring of health at home. Time pressures placed on parents due to caregiving responsibilities often led to needed, but not urgent, services being missed. CH may aid access in this regard through reducing the impacts of logistics such as time and travel.

CH may also reduce illness-related barriers to accessing services. Treatment regimens may impact the immune system, requiring isolation to reduce the risk of infection. While in-person services may be unavailable, CH was seen to allow continued access to services while in isolation. The ability to monitor health outcomes from home was seen to offer families the opportunity to engage more within their communities. Due to the impacts of some paediatric cancer treatments on the immune system, parents were often hesitant to attend events. Through real-time monitoring of child health, parents could be more aware of their child’s immune system and thus more confident to engage in activities. CH was also noted as a potential avenue through which children could access peers with children noted to be eager to engage socially online.

##### Decrease access

Both groups noted the significant financial pressure imposed by a paediatric cancer diagnosis. Additional cost for CH may further add to this. Parent literacy may also prevent access to CH, with poor literacy felt to be common, particularly for at-risk groups. The absence of strong WI-FI connectivity across Ireland and within hospitals may limit CH use. Further pressure to access high-quality WI-FI signals to manage their child’s care was thought to present additional burden for parents.

## Discussion

This study sought to explore HCP and parents’ perspectives on the potential use, barriers and facilitators of CH, to better understand how its uptake may be facilitated in practice. Areas of need that may be supported by CH include communication, individualisation of care and access to information. Consideration to the importance of trust, pace of change and impact of digitalisation on access to services was also highlighted. These results suggest several roles for CH in paediatric cancer, including supporting access to services, individualised treatment, illness monitoring, aiding communication between stakeholders, reducing administrative and decision burden from parents, and meeting informational needs. However, the pace of digitalisation appears slow and hard fought, with concerns regarding privacy and digital skills raised. It is of note that the present analysis was conducted in the context of a healthcare system within which digitalisation efforts have been slow and limited in scope. As such, while the utility of CH broadly was explored, many of the needs raised could likely be addressed with simpler technological solutions such as EHRs. While results suggest acceptance and enthusiasm by key stakeholders towards the use of CH, barriers should be considered to ensure effective implementation.

Parent and HCP willingness to use CH to support care is consistent with Sin et al. [50], who found good acceptance of eHealth psychosocial interventions for family caregivers. As acceptance is a predictor of use [22], these results are positive and suggest a willingness to engage with CH. The positive attitudes noted by HCPs too are promising and may support CH use due to the known impact of HCPs on patient attitudes [27]. While primarily positive attitudes were noted, several concerns were also raised regarding data privacy, which may inhibit CH use. This is of note as, within the TAM, data privacy has been found to impact acceptance [38]. The broader study context, however, may have impacted these results. As data breaches in healthcare have occurred within the Irish health service in recent years [51], privacy concerns and distrust in security are somewhat expected. The importance of ensuring data security in CH has been noted across health sectors [52], with a clear need for robust regulatory and privacy frameworks [53]. As personal data protection is a right within Europe [54], ensuring privacy within any CH tool is of the utmost importance. While results are promising, additional effort to address privacy concerns is needed to facilitate CH use in practice.

The present analysis highlights several avenues through which CH may support families impacted by paediatric cancer. One means is through the sharing of information, both across healthcare teams and between HCPs and parents. Shared access to information across HCPs and parents may decrease parental responsibility, while simplifying information sharing may enable enhanced communication between parents and HCPs. This mirrors previous research suggesting the potential for CH to support communication in paediatric cancer [13], as well as communication between primary and secondary care [9] and sharing of clinical information [10]. However, this need for increased access to health information may be facilitated by more simple CH approaches, such as the use of EHRs, which are unavailable in Ireland. While communication was felt to be an area positively impacted by CH, concerns were raised regarding reductions in face-to-face supports, which is consistent with the previous research [55]. As such, while CH may be beneficial in supporting communication between key stakeholders, efforts to ensure it does not replace in-person communication are needed, along with additional analysis of the most optimal frequency and form of communication between parents and HCPs to ensure effective distribution across communicative modalities. Further, in the context of the low digitalisation encountered by this population, analysis of the impacts of more simple CH technologies on communication and information needs is required to determine whether these may sufficiently meet needs.

Another clear finding from this study was parental informational needs. Support in seeking, sharing and managing information were all felt to be areas in which CH may provide support, which is consistent with past research finding how CH can support parent understanding of child health [12]. The importance of reliable health information is particularly necessary in the context of the negative impacts of misinformation on treatment adherence, inappropriate treatment seeking [56] and patient–HCP relationships, alongside the difficulties in falsifying misinformed beliefs [57, 58]. CH, however, may mitigate these impacts through the use of machine learning to remove such health misinformation [59]. As such, CH may be beneficial in establishing trustworthy and accurate sources of health information, mitigating these concerns. In the context of the TAM, CH may be perceived as useful through easing access to health information. This in turn may positively impact acceptability. For HCPs too, CH had perceived benefits in increasing access to information on child responses to treatment. More specifically, means to monitor health through digital technologies were felt to impact positively on responses to infection, health outcomes and time in clinical settings. For parents, the importance of a reliable source of information was noted, with a need for technology to be sourced from a trusted health professional. The key role of HCPs in the dissemination of digital technologies has been found previously with a need for CH to be integrated within care pathways [60]. As such, while results suggest information provision as an area in which CH may provide support, efforts are needed to aid HCPs in the dissemination of technologies to parents of children with cancer to support uptake.

The absence of digitalisation and pace of change within the healthcare service in Ireland was described as a key barrier to the use of CH within paediatric cancer. The healthcare service was seen to be reluctant to introduce digital technologies. Organisational reluctance to change and ineffective change management have been found to be key impediments to the use of CH [10]. This lack of digitalisation is an area of focus within healthcare in Ireland, with the national eHealth strategy advocating for digitalisation as a national infrastructural investment [40]. Specifically, there is a need for a properly executed national eHealth strategy, with an emphasis on delivery of key areas of digitalisation such as ePrescription and digital medical records, amongst others. Reluctance to digitalise care in Ireland may be impacted by past unsuccessful digitalisation efforts, such as electronic voting and PPARS (Personnel, Payroll And Related Systems) resulting in reluctance to trust technology-based interventions [61]. It is also of note that this study was completed prior to the ransomware attack of Irish hospitals in June of 2021, which resulted in the loss of IT systems and breaches of personal healthcare data [51]. This event may have impacted trust in CH and willingness of the healthcare system to further invest in digital technologies. Organisational factors play a significant role in the use of CH with additional efforts needed to examine how best to support effective and timely change to ensure technologies are effectively employed.

Several limitations to the present study are noted. Firstly, the timing of the study may serve as a limitation, with interviews conducted during the COVID-19 pandemic with restrictions on non-essential movements across the country. Due to social distancing requirements, many previously in-person services were moved online. This increased access to digital health services may have impacted the perceived acceptability of CH through demonstrating its use in practice. Many respondents noted the impact of COVID-19 on their use of technology and the opportunity it presented to trial digital service delivery in previously hesitant areas. The absence of digitalisation within the Irish healthcare system also poses a limitation, as some needs identified may be addressed through the adoption of technologies such as EHRs, which are highly prevalent across healthcare services globally [62], but not available in Ireland. This in turn limits our ability to explore more complex connected health technologies, as basic digitalisation remains outstanding. A further limitation is the small sample size and constituents. However, as good variety in experiences for both parents and HCPs were obtained, this may have allowed for a broader range of views to be captured. As few males were included, further analysis of the perspectives of this group are needed. Additionally, the voice of children themselves was omitted from the present study. As many CH tools in the space are targeted towards parents rather than children, their participation was not sought. To determine perspectives on the utility of CH for children themselves, particularly teenagers or older children who may derive increased agency over own health information through such tools, additional analysis is needed.

The present analysis highlights several practical considerations requiring further analysis. While results suggest positive attitudes towards CH for both HCPs and parents, the slow pace of digitalisation remains a concern, with efforts needed on the part of the healthcare system. Technology developers should consider the financial, technological and skill requirements when creating CH interventions. The results of the present study cast positive light on the potential for CH to be effectively employed to support families impacted by paediatric cancer. The need for inclusion of parents, HCPs and the broader healthcare systems within the design of CH in user-centred design is clear to ensure alignment between technological advances and service delivery. While further analysis is need on the efficacy of CH to support families impacted by paediatric cancer, the present findings highlight key areas where CH may be effectively employed.

## Supplementary Information

Below is the link to the electronic supplementary material.

Supplementary file1 (DOCX 16 KB)

## Data Availability

To ensure the confidentiality of participants, particularly within an Irish context in which there are few parents of children with cancer and few HCPs, data are not freely available.

## Code availability

N/A.
