# Supplementary material for: Utility, barriers and facilitators to the use of connected health to support families impacted by paediatric cancer: a qualitative analysis
Source: Support Care Cancer. 2022 May 6;30(8):6755–66. doi: 10.1007/s00520-022-07077-4 (PMC9075925; doi:10.1007/s00520-022-07077-4)
Supplement: Supplementary file 1 — Supplementary file1 (DOCX 16 KB) [file 520_2022_7077_MOESM1_ESM.docx]

Interview Questions – Parent/caregivers

Parent/Caregivers Demographic Information:

| Parent Age |  |
| --- | --- |
| Parent Gender |  |
| Parent Marital Status |  |
| Parent Ethnicity |  |
| Child Gender |  |
| Child Age |  |
| Child Diagnosis |  |
| Time since active treatment |  |
| Family size |  |
| Travel distance to treatment centre |  |
| Needs met? | Service Needs Y**/**N   -  Mental Health Y/N  Financial Advice Y/N |

 Interview Questions:

| 1) Unmet needs of families impacted by paediatric cancer | What do you feel are the needs, challenges, and currently  available supports for parents, children and families impacted by paediatric cancer? |
| --- | --- |
|  | Do you feel these needs are met? If not, why? |
|  | What additional services do you feel are needed? |
| 2) Attitudes towards Connected Health | Connected health is defined as the use of smart technologies, like sensors, telehealth or electronic health records, within healthcare. It differs from other technologies in that a two-way flow of information is used. Information is gathered, analysed and then fed back to the individual.    With that in mind what potential use would CH offer parents, children and families impacted by paediatric cancer? |
|  | What currently unmet needs of parents, children and families affected by paediatric cancer could CH support? |
|  | What barriers or limitations would there be to the use of CH? |
| 3) Analysis of the  impact of  the therapeutic  recreation based hospital outreach program: | What do you feel was your child’s experience of the camp programme and if any subsequent impact following  attendance was noted. |
|  | What do you feel are the core features of the HOP? |
|  | How could HOP be improved in future? |
|  | What impact do feel the following had on your Childs experience of the HOP?   1. Social Support 2. Therapeutic landscapes – defined as the creation of healing sense of place within an environment 3. Opportunity to have a break 4. Opportunity to engage in leisure activities |
|  | How do you feel about the use of technology in HOP to date? |
|  | What impact do you feel HOP had on your children’s self-esteem? |
